# Perception of a caring ethical climate as a factor facilitating the stress and exhaustion of employees with dark triad traits of personality: a moderated mediation model

**DOI:** 10.3389/fpsyg.2026.1768371

**Published:** 2026-05-21

**Authors:** Marcin Wnuk

**Affiliations:** Department of Work and Organisational Psychology, Faculty of Psychology and Cognitive Science, Adam Mickiewicz University, Poznań, Poland

**Keywords:** burnout, caring ethical climate, dark triad traits, job stress, machiavellianism, narcissism, psychopathy

## Abstract

**Objective:**

A good match between employees and the organization they work for is beneficial for both parties; however, a misfit between the two can lead to harmful effects. There is a noticeable absence of research showing that a misfit between workers characterized by higher levels of dark triad traits and workplace ethical standards has negative consequences. This study is an attempt to fill this gap by verifying whether the perception of a caring ethical climate by employees scoring higher on dark triad traits may facilitate their job stress and exhaustion.

**Method:**

The study was cross-sectional. The participants were 1,000 employees from Poland who had been employed by their current company for at least 1 year.

**Results:**

Perception of a caring ethical climate moderated the relationship between dark triad traits of personality and job stress as well as exhaustion. Also, the indirect effects of the dark triad traits of narcissism, Machiavellianism, and psychopathy on exhaustion through job stress and moderated by the perception of a caring ethical climate were found. The hypothesis about detrimental consequences of a misfit between workers scoring higher on narcissistic, Machiavellian, and psychopathic, and a caring ethical work climate were supported.

**Conclusions:**

The study showed that adaptation to the requirements of a caring ethical climate of an organization, which are antagonistic to preferences of employees possessing a higher level of dark triad traits, is associated with emotional costs for these employees. The perception of a caring ethical climate by employees scoring higher on dark triad traits facilitates their stress and exhaustion. The practical implications of the study's outcomes are discussed, emphasizing the need to match ethical preferences between potential employees and the organization during recruitment.

## Introduction

Burnout manifests as a serious mental and emotional problem that influences job performance, engagement, and satisfaction, and it often requires psychological intervention and generates economic and healthcare costs. Only nine European Union countries, which do not include Poland, recognize burnout syndrome as an occupational disease, and in four of these countries compensation for burnout syndrome may be provided (Lastovkova et al., 2018). According to research conducted on a sample of 1,080 Polish workers, 78% experienced at least one symptom of burnout UCE Research and ePsycholodzy.pl (2024).

The World Health Organization's approach to burnout was inspired by and based on the definition of burnout by Maslach et al. (1996) as “a state of exhaustion in which one is cynical about the value of one's occupation and doubtful of one's capacity to perform” (p. 20). It was incorporated into the *International Classification of Diseases* (ICD-11), as a phenomenon exclusively related to the work domain of life, consisting of “(1) feelings of energy depletion or exhaustion; (2) increased mental distance from one's job, or feelings of negativism or cynicism related to one's job; and (3) a sense of ineffectiveness and lack of accomplishment” (World Health Organization, 2019, 2022).

The operationalization of burnout has evolved, resulting in 13 different definitions (Guseva Canu et al., 2021); however, a common core of these definitions reflects two main characteristics: (1) the *inability* to invest effort in work (exhaustion) and (2) a lack of *willingness* to do so (distance) (Demerouti and Bakker, 2025; Schaufeli and Taris, 2005).

An inherent element of burnout is chronic and prolonged job stress (World Health Organization, 2019, 2022), which may have various antecedents, including the employee's personality (Chang, 2009) and their susceptibility to perceiving work events as threats or losses (Lazarus and Folkman, 1984). Personality, as one of the individual sources of stress and exhaustion (Chang, 2009), is rarely discussed in the literature, especially using a person-situation interactionist model (Mischel and Shoda, 1995) which encompasses the perception of the organizational environment by employees with dark triad traits.

Only a few studies have tested the potential mechanisms underlying the link between the dark triad traits, such as narcissism, Machiavellianism, and psychopathy, and burnout. For example, Ma et al. (2021) reported a negative relationship between narcissism and exhaustion in a U.S. sample and an irrelevant association in a Chinese sample. Similarly, Machiavellianism correlated positively with exhaustion in the United Kingdom, but this relationship was non-significant in China, suggesting a moderating role of national cultures in the links between the dark triad traits and exhaustion. Barnett and Flores (2016) confirmed that self-compassion mediates the link between narcissism and school burnout. Similarly, Johnson et al. (2015) found that secondary psychopathy can be related to less supervisor support, which leads to increased emotional exhaustion.

Previous studies have not considered transactional factors that reflect how personality (the dark triad traits) interacts with organizational factors (work demands, role ambiguity) to explain burnout (Chang, 2009). It is essential to determine whether employees who score higher on dark triad traits dark triad traits perceive ethical organizational standards in a manner that has a beneficial or detrimental effect on their stress and exhaustion. This is important because of the need, first, to understand whether a caring ethical climate will favor this group of workers or protect them from exhaustion. Second, it has practical implications regarding the fit between employees with dark triad traits and the ethical climate within the recruitment process (Kristof-Brown et al., 2023).

This study aims to fill this gap by examining the potential interaction between dark triad personality and the perception of a caring ethical climate in explaining exhaustion, both directly and indirectly, through job stress. In other words, this study focuses on verifying the mechanisms underpinning the relationship between the dark triad traits and exhaustion, considering the mediating function of stress and the moderating role of a caring ethical climate. The theoretical ground of this research is person-organization fit theory (P-O fit; Kristof-Brown et al., 2023) and trait activation theory (Tett and Burnett, 2003). Following the P-O fit approach, a misfit between characteristics of employees with a higher level of narcissism, Machiavellianism, and psychopathy and the requirements and expectations resulting from the perception of a caring ethical climate may lead to more stress and exhaustion. According to trait activation theory, the stronger the cues and incentives they perceive in a caring ethical climate, the more likely they are to feel encouraged to behave in a manner inconsistent with their needs and values, which may lead to increased stress and exhaustion.

P-O fit theory describes the relationship between employees and organization from the mutual suitability point of view, distinguishing between two types of fit: complementary and supplementary (Kristof-Brown et al., 2023). Complementary fit refers to the situation in which an organization or employee possesses characteristics that are desirable and required by the second party. It can be the set of special skills that the worker possesses but is lacking and required by the organization, or the organization may offer work conditions that are crucial for the employee, such as guaranteeing autonomy, power, status, influence, or a competitive salary.

Supplementary fit encompasses the similarities between certain characteristics of the employee and the organization, meaning that the employee and the organization may share values, needs, goals, and so on. The general rule, based on research outcomes, is that a P-O fit yields fruitful and beneficial outcomes (Kristof-Brown et al., 2005; Verquer et al., 2003), whereas a P-O misfit has harmful effects (Englert et al., 2023).

Trait activation theory describes the interactions between employees characterized by certain personality traits and work environment requirements (Tett and Burnett, 2003). This theory posits that employees perceive signals from the work context, which are interpreted as cues and incentives to elicit appropriate behaviors. In other words, the situational context in the workplace, to a significant extent, determines the worker's attitude, acting as a trigger to manifest certain personality traits. The probability of evoking a particular behavior depends on the strength of the signals that the employees receive.

## Review of literature

### Dark triad and job stress and burnout

The dark triad traits of narcissism, Machiavellianism, and psychopathy have a common root consisting of antisocial characteristics such as problems with empathy (Dinić et al., 2021; Shukla and Upadhyay, 2025), lack of agreeableness (Dinić et al., 2021; Kaufman et al., 2019), selfishness (Dinić et al., 2021; Wnuk, 2025), aggressiveness (Dinić et al., 2021), exploitation of others (Kajonius et al., 2016; Knitter et al., 2025), tendency to rivalry (Trahair et al., 2020), and desire for power, status, and influence (Jonason and Ferrell, 2016; Kajonius et al., 2015; Lee et al., 2013). Research has confirmed the self-centered attitude and pursuit of self-investment of individuals who scored higher on dark triad traits, showing that this group of workers behaves more egoistically at work (Wnuk, 2025). They are oriented on self-enhancement values, such as achievement and power, and they want to lead a hedonistic life (Kajonius et al., 2016; Kaufman et al., 2019).

The outcomes regarding the relationship between the dark triad traits with stress and burnout are inconsistent and depend on sociocultural context, research sample, and measures applied for verification of these constructs. Generally, Machiavellianism and psychopathy mostly go hand in hand with job stress and exhaustion, but the findings regarding correlations of these constructs with narcissism are negative, positive, or irrelevant.

For instance, in Spanish and Portuguese samples, narcissism, Machiavellianism, and psychopathy were positively related to stress and anxiety, regardless of sex (Jonason et al., 2022). Also, the psychopathological role of Machiavellianism and narcissism was identified among German adults (Hornstein et al., 2025). Machiavellianism positively predicted depression, anxiety, and stress, but narcissism only predicted stress. Psychopathology was irrelevant for all psychopathological symptoms. Birkás et al. (2020) demonstrated that stronger Machiavellianism is associated with greater perceived distress. Inverse correlation was noted between narcissism and distress perception. In a longitudinal study conducted by Wei et al. (2025), narcissism negatively predicted later psychopathology consisting of depression, anxiety, and stress symptoms and was unrelated to stress at two of the three points of time. Psychopathy and Machiavellianism positively correlated with stress at all three time points.

For example, among Slovakian teachers, all the dark triad traits were positively related to emotional exhaustion (Copková, 2021). Bradić et al. (2025) showed that emotional exhaustion was positively related to the antisocial behavior aspect of psychopathy and to the cynical view of human nature aspect of Machiavellianism but was irrelevant for narcissism.

### Ethical climate

The ethical climate encompasses the shared perception of the established standard of ethical conduct within an organization, influencing employee decision-making and attitudes in the workplace **(**Victor and Cullen, 1988). It serves as a compass that guides what behavior is considered appropriate and acceptable and what is undesirable and stigmatized from an ethical point of view. Among the five types of organizational ethical standards empirically distinguished by Victor and Cullen (1988), two are antagonistic, namely, caring and instrumental (Parboteeah et al., 2024). A caring ethical climate is oriented toward the well-being of every employee, with the organization's primary concern being to support the achievement of this **(**Victor and Cullen, 1988). In this type of climate, values that are appreciated are altruism, support, cooperation, compassion, friendship, empathy, and benevolence, as well as a focus on affiliation needs. Conversely, within an instrumental climate, the well-being of the individual takes precedence, even at the expense of the community **(**Victor and Cullen, 1988). This type of climate emphasizes the importance of individual achievements, attaining power and status, competitiveness, and the pursuit of self-centered desires, aspirations, and goals (Bates et al., 2025).

Research outcomes suggest that a caring ethical climate is considered positive in an organization, while an instrumental ethical climate is treated as negative (Bates et al., 2025). The perception of a caring ethical climate may have beneficial outcomes, such as organizational commitment, job satisfaction, performance (Parboteeah et al., 2024; Wnuk et al., 2025a), organizational identification (Teresi et al., 2019), organizational pride (Wnuk et al., 2025a), organizational safety (Li and Peng, 2022), and prevention of dysfunctional behavior (Parboteeah et al., 2024) such as organizational cynicism (Wnuk et al., 2025a), emotional exhaustion (Li and Peng, 2022), turnover intention, and workplace bullying (Freire and Pinto, 2022). On the other hand, the perception of an instrumental climate favors moral distress (Zare-Kaseb et al., 2025), job role stress (Zhang and He, 2024), emotional exhaustion (Saleh et al., 2023), turnover intention (Saleh et al., 2023), antisocial behavior (Parboteeah et al., 2024) such as egoism at work (Wnuk, 2025), and organizational cynicism (Wnuk et al., 2025b) and is negatively related to organizational commitment, job satisfaction (Parboteeah et al., 2024; Wnuk et al., 2025b), and organizational pride (Wnuk et al., 2025a). For example, Polish workers who perceived their organization's ethical climate as caring were more likely to find meaning in their work, experienced less stress, and were less motivated to leave the organization (Wnuk et al., 2025a). On the other hand, the perception of an instrumental climate was positively related to stress, turnover intention, and the manifestation of symptoms of existential frustration at work (Wnuk et al., 2025a).

## Research justification

Previous research has found an instrumental ethical climate to be favorable for employees scoring higher on dark triad traits and negatively correlated with the perception of a caring ethical climate (Wnuk et al., 2025a,b). Wnuk's study (2025) found that an instrumental climate, due to its individualistic and egocentric requirements, is desirable and is well suited for employees with the higher level of dark triad traits as it is a supplementary fit to their values and needs (Kristof, 1996), providing them with the opportunity to satisfy their selfish ambitions and aspirations.

Earlier studies considered selfishness a strong motivational factor of employees characterized by higher level of dark triad traits. The self-orientation among these workers was supported by Raine and Uh's (2019) study, in which egocentric, adaptive, and pathological forms of selfishness were positively associated with narcissism, Machiavellianism, and psychopathy. Additionally, in a sample of Polish youth, generational egoism was positively associated with the dark triad traits (Próchniak et al., 2025). In the same vein, Deutchman and Sullivan (2018) reported a stronger likelihood of individuals scoring higher on dark triad traits behaving selfishly in the Prisoner's Dilemma. These outcomes align with Jonason et al.'s (2010) findings, which confirmed that the agentic social style is preferred by individuals with higher levels of dark triad traits, who build social relationships based on an individualistic and competitive approach. In the same vein, Kiral Ucar et al. (2023) demonstrated that these individuals have a propensity for an egoistic value orientation and an aversion to an altruistic value orientation.

On the other hand, a caring ethical climate, as opposed to an instrumental one, may not be suited to individuals with the dark triad traits personality due to differing social environment standards. Instead of altruism, cooperation, harmonious relationships with workmates, support, agreeableness, and empathy, workers who score higher on narcissism, Machiavellianism, and psychopathy may display selfishness, confrontation (Jonason et al., 2010), exploitation of others (Kajonius et al., 2016; Knitter et al., 2025), and aggressiveness (Dinić et al., 2021). This can make behaviors expected of them inconsistent with their predispositions and tendencies, resulting in increased stress and exhaustion. The pursuit of power in a selfish and competitive manner, guided by the principle that the end justifies the means, may necessitate confronting numerous obstacles, evoking necessity adapting to unwanted workplace ethical expectations and changing attitudes. This can generate anger, disappointment, frustration, and tension, especially when confronting an undesirable organizational standard of conduct that requires the use of a completely different set of behaviors than those favored by these employees.

Earlier studies have reported that individuals who score higher on dark triad traits are more likely than other employees to behave in accordance with the organizational context, particularly when it aligns with their needs and values. Among Polish workers, perception of an instrumental ethical climate amplified the positive relationship between psychopathy and egoism at work (Wnuk, 2025). Additionally, Webster and Smith (2019) demonstrated the beneficial effect of supplementary fitness on the relationship between the dark triad traits and the work environment. They confirmed that the perception of a high-involvement management climate reduces the negative effect of Machiavellianism on organizational citizenship behaviors. O'Boyle et al. (2012) found that being in a role of authority weakened the negative association between psychopathy and counterproductive work behavior.

On the other hand, individuals with higher levels of dark triad traits are sensitive to external sources of motivation (Prusik and Szulawski, 2019), even if they do not align with their agentic social style (Bereczkei et al., 2010; O'Boyle et al., 2012) and demand a prosocial attitude. In such situations, a lack of fit between their preferences and organizational standards has a harmful effect, or they pretend to act prosocially in accordance with social expectations. For example, in O'Boyle et al. (2012), a stronger perception of ingroup collectivism was associated with weaker job performance among narcissistically inclined employees, implying that the prosocial orientation of these workers may harm their work effectiveness. Bereczkei et al. (2010) found that Machiavellian-oriented individuals use tactics that rely on pretending to be altruistic when observed by others but revert to a selfish strategy when they are not observed.

Given that a misfit between employees and the ethical environment (Kristof, 1996) may lead to harmful effects, I assume that the aversion of employees with higher level of dark triad traits to prosocial behaviors, which are required and appreciated in a caring ethical climate, along with their stronger perception of this kind of climate and need to adapt to social expectations inconsistent with their self-interested approach, may evoke a feeling of inauthenticity and will boost these employees' stress and emotional exhaustion. In addition, the negative effects of narcissism, Machiavellianism, and psychopathy will be elevated in parallel with a stronger perception of a caring ethical climate, despite the fact that this kind of ethical climate is expected to protect employees from stress and exhaustion (Li and Peng, 2022; Wnuk et al., 2025b). Previous research has consistently demonstrated that the perception of a caring ethical climate, which is not desired by employees who score higher on dark triad traits, may be protective against stress and exhaustion for most employees, but not in this group of workers, in contrast to an instrumental climate, which is preferred by them but results in stress and exhaustion for most employees (Li and Peng, 2022; Saleh et al., 2023; Wnuk et al., 2025b; Zare-Kaseb et al., 2025; Zhang and He, 2024).

This study had two hypotheses:

H1: In a sample of Polish employees, the perception of a caring ethical climate moderates the relationship between the dark triad traits (narcissism, Machiavellianism, psychopathy) and job stress, strengthening this positive relationship.
H2: In a sample of Polish employees, the perception of a caring ethical climate moderates the relationship between the dark triad traits (narcissism, Machiavellianism, psychopathy) and exhaustion, strengthening this positive relationship.

I also hypothesized that the dark triad traits are indirectly and positively related to exhaustion through the potential elevation of stress amplified by the stronger perception of a caring ethical climate. Experiencing chronic stress is a pivotal antecedent of exhaustion, which is supported by a plethora of studies (McManus et al., 2002; Ronen and Baldwin, 2010; Schwarzer and Hallum, 2008; Ulaş and Seçer, 2025; Xie et al., 2024; Xu and Wang, 2023). For example, McManus et al. (2002) demonstrated, in their longitudinal study among U.K. doctors, the reciprocal nature of the relationship between stress and emotional exhaustion. They found that higher levels of stress caused stronger emotional exhaustion, which in turn secondarily increased stress. In the same vein, Schwarzer and Hallum (2008) reported that stress mediated the relationship between teacher self-efficacy and burnout in both cross-sectional and longitudinal research designs. Finally, Ronen and Baldwin (2010) confirmed that attachment anxiety influences burnout prospectively through increasing stress.

H3: In a sample of Polish employees, job stress positively predicts exhaustion.
H4: In a sample of Polish employees, job stress mediates the relationship between the dark triad personality traits (narcissism, Machiavellianism, psychopathy) and exhaustion, and this indirect link is moderated by the perception of a caring ethical climate, strengthening this positive indirect relationship.

## Method

### Sample

The distribution of sociodemographic variables is presented in Table 1. The study's participants were 1,000 workers with employment contracts. The sample consisted of slightly more males (54.9%) than females (45.1%), with a mean age of 41.06 years (*SD* = 10.77). Almost half of the participants (45.7%) had an educational level above high school. The mean length of employment with the current employer was 9.41 years (*SD* = 8.59), while the mean length of employment overall was 17.29 years (*SD* = 10.79). The majority were employed in companies with at least 50 employees (59.2%). At least one in four workers held a managerial position (23.6%).

**Table 1:** Sample characteristics (*N* = 1,000).

Variable	*n* (%)
Sex	
Men	549 (54.9)
Women	451 (45.1)
Age (in years)	41.06 (10.76)^a^
Marital status	
Single	211 (21.1)
Married	550 (55)
Informal relationship	136 (13.6)
Informal relationship (living separately)	42 (4.2)
Widower	15 (1.5)
Divorced	53 (5.3)
Separated	1 (0.1)
Number of children	1.16 (1.10)^a^
Number of dependents	1.67 (1.72)^a^
Education level	
Primary	11 (1.1)
Lower secondary	5 (0.5)
Vocational	95 (9.5)
High school	432 (43.2)
Post-secondary	447 (44.7)
Ph.D.	10 (1.0)
Total work experience (in years)	18.77 (48.11)^a^
Form of employment	
Full-time contract	1,000 (100)
Seniority in present work (in years)	9.40 (859)^a^
Sector of employment	
Public	663 (66.3)
Private	326 (32.6)
Non-governmental organization	11 (1.1)
Organization size	
1–9 employees	135 (13.5)
10–49 employees	273 (27.3)
50–249 employees	258 (25.8)
250 employees and more	334 (33.4)
Managerial position	
No	767 (76.7)
Yes, lower-level manager	109 (10.9)
Yes, a middle-level manager	93 (9.3)
Yes, a higher-level manager	31 (3.1)

^a^ For continuous variables means and standard deviations (in parenthesis) are presented.

### Procedure

The study protocol was approved by the Ethics Committee at the [masked for review]. The data were collected by an organization specializing in this kind of project through the Manulo research panel. The criteria for participation were being an adult, living and working in Poland, possessing a contract of employment in a full-time job, and having worked for the current organization for more than 1 year.

All participants provided written online consent to take part in the research. They were advised of the anonymity of their responses and had the option to withdraw from the study at any time without consequences.

### Measures

#### Dark triad

For the verification of dark triad personality traits, the Polish version of the Dirty Dozen scale was used (Czarna et al., 2016). This measure consists of three factors—narcissism, Machiavellianism, and psychopathy—each with four items with responses on a 5-point Likert scale (ranging from 1 = *strongly disagree* to 5 = *strongly agree*). This tool exhibits satisfactory psychometric properties for the Polish population, specifically in terms of internal consistency and test–retest reliability.

#### Caring ethical climate

A caring ethical climate was examined using four questions that serve as indicators in the Polish version of the Ethical Work Climate Questionnaire (Wnuk et al., 2025a). A caring ethical climate is one of the five types of ethical climates distinguished empirically by Victor and Cullen (1988), authors of the Ethical Work Climate Questionnaire. Answers are provided on a 5-point Likert scale (from 1 = *strongly disagree* to 5 = *strongly agree*). The Polish version of the Ethical Work Climate Questionnaire was tested for internal consistency, divergent and convergent validity, and reliability, achieving satisfactory results (Wnuk et al., 2025a).

#### Job stress

Job stress was examined using a single-item measure. Participants responded to one question, “In general, how do you find your job?” with five possible options: (1) not at all stressful, (2) mildly stressful, (3) moderately stressful, (4) very stressful, and (5) extremely stressful (Smith, 2000). This single-item measure is an appropriate and acceptable indicator of overall job stressfulness (Houdmont et al., 2021).

#### Exhaustion

Burnout was measured by the Polish version of the Oldenburg Burnout Inventory (OLBI; Baka and Basińska, 2016; Demerouti et al., 2003). The OLBI consists of two factors: disengagement and exhaustion. The Polish version of the OLBI presents good psychometric properties in terms of reliability, internal validity, and discriminant validity. Four negative items related to exhaustion were selected. Research participants answered on a Likert scale rated from 1 (*agree*) to 4 (*disagree*).

### Statistical analysis and preliminary results

Harman's one-factor test was applied to assess the possibility of common method bias. Exploratory factor analysis revealed that one factor explained 35.09% of the variance, which is less than the 40% threshold, indicating that the data were free from common method bias (Podsakoff et al., 2003). Additionally, the common latent factor method within confirmatory factor analysis was conducted, which showed poor fit (χ^2^: 6,571.228; χ^2^/df: 34.76; *p* < 0.01; CFI: 0.55; NFI: 0.54; SRMR: 0.17; RMSEA: 0.18), supporting the absence of common method bias. To identify a potential multicollinearity problem, variance-inflated factors were estimated for the research model of regression. Each predictor has values significantly less than the arbitrary norm of 10 (see Table 2) (O'Brien, 2007), which means that the research data are not contaminated by multicollinearity. In addition to the alpha Cronbach's coefficient, composite reliability was computed, exceeding the 0.7 threshold for all research variables (see Table 2) (Fornell and Larcker, 1981). Also, following the Fornell and Larcker (1981) criteria for discriminant validity, the average variance extracted exceeded the established 50% standard for narcissism, Machiavellianism, and exhaustion, and was close to this standard for psychopathy (see Table 2).

**Table 2:** Descriptive statistics (*N* = 1,000).

Variable	Minimum	Maximum	Mean	Standard deviation	Skewness	Kurtosis	VIF	AVE	CR	Cronbach's alpha coefficient
Narcissism	4	20	8.96	4.22	0.42	−0.85	3.19	72.81	0.91	0.91
Machiavellianism	4	20	7.78	4.00	0.79	−0.47	3.06	72.70	0.92	0.91
Psychopathy	4	20	8.22	3.63	0.62	−0.42	8.22	49.40	0.79	0.80
Exhaustion	4	16	10.89	2.93	−0.12	−0.33	–	68.52	0.88	0.86
Caring ethical climate	0	20	10.99	4.83	−0.44	−0.02	1.02	78.65	0.94	0.92
Stress at work	1	7	4.43	1.45	−0.28	−0.22	1.02	–	–	–

VIF, variance inflation factor; AVE, average variance extracted; CR, composite reliability.

**Figure 1:**
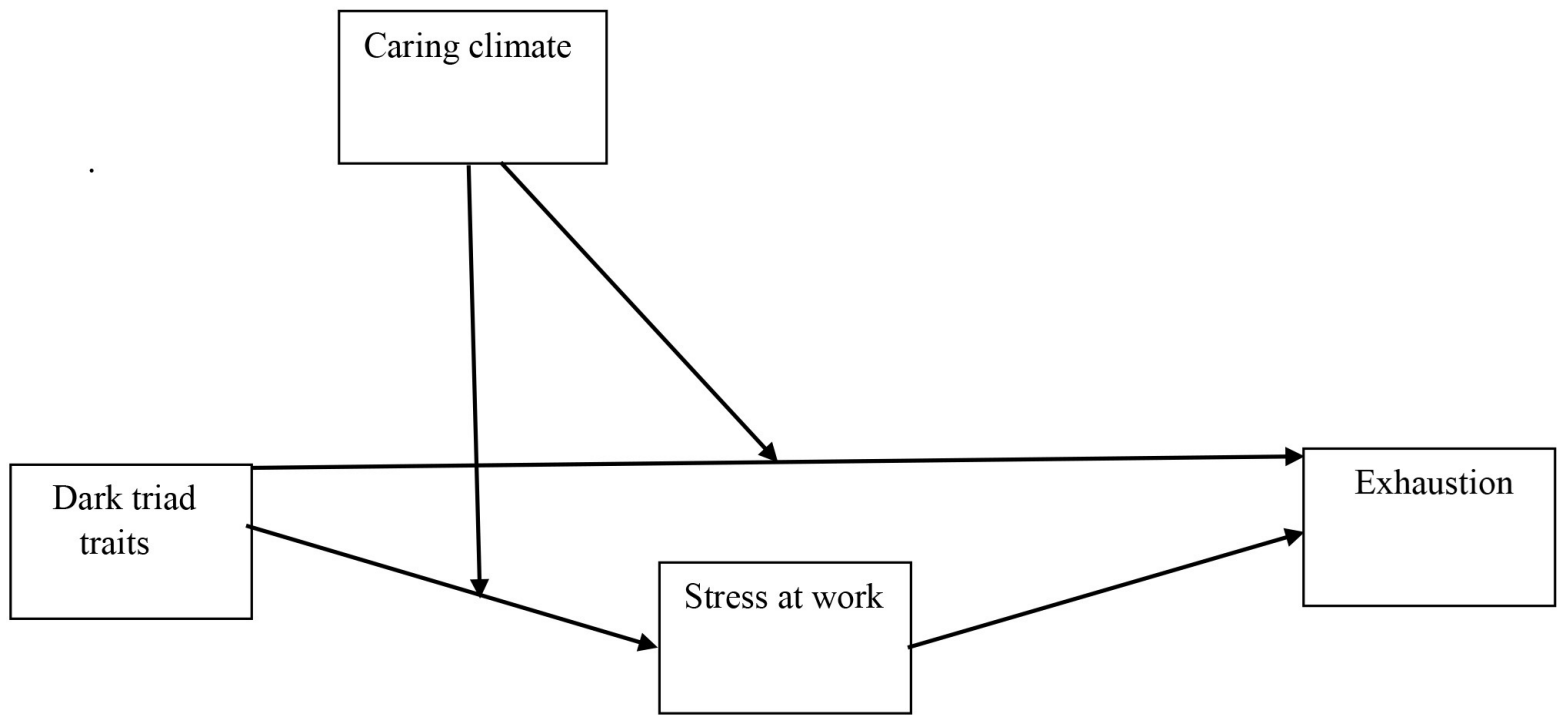
Theoretical model.

The study model was examined using PROCESS macro software (Preacher and Hayes, 2004). The bootstrapping method indicates indirect and moderating effects, as evidenced by whether the bias-corrected 95% confidence interval (CI) includes zero (Preacher and Hayes, 2004). For this, Model 8 with bootstrapping using a 5,000 subsample was employed, which accurately reflected the theoretical research model. In line with Hayes's (2013) recommendations, if the Boot LLCI and Boot ULCI do not contain zero, this confirms statistically significant effects between the variables. In Model 8, particular dark triad traits were independent variables; the perception of a caring ethical climate was a moderator between dark triad traits and stress, and stress was a mediator between dark triad traits and exhaustion (see Figure 1). Based on a review of the literature, to the research model was introduced with sociodemographic variables identified in previous research as potential predictors of burnout, such as sex (Artz et al., 2022), age (Schaufeli and Enzmann, 1998), education level (Maslach et al., 2001), total work experience, and seniority in the present work (Brewer and Shapard, 2004). In every regression model, the particular dark triad trait was included, and other dark triad traits were introduced as covariates.

The values of the correlation coefficients between the study variables are presented in Table 3. Narcissism, Machiavellianism, and psychopathy were positively intercorrelated. None of them were correlated with exhaustion, and only Machiavellianism was positively correlated with stress. A negative relationship between the perception of a caring ethical climate and stress as well as exhaustion and a positive relationship with narcissism were found. The links between Machiavellianism and psychopathy with the perception of a caring ethical climate were irrelevant.

**Table 3:** Values of r-Pearson correlation coefficients between research variables (*N* = 1,000).

	1	2	3	4	5
1. Narcissism					
2. Machiavellianism	0.74^**^				
3. Psychopathy	0.66^**^	0.82^**^			
4. Exhaustion	0.02	0.05	0.02	.	
5. Caring ethical climate	0.12^**^	0.04	0.03	−0.29^**^	
6. Job stress	0.03	0.12^**^	0.05	0.38^**^	−0.06^*^

^*^p < 0.05.

^**^p < 0.01.

### Hypotheses verification

Hypothesis 1, regarding the moderating role of the perception of a caring ethical climate in the relationship between the dark triad personality traits and stress, was supported. Narcissism, Machiavellianism, and psychopathy, in interaction with perception of a caring ethical climate, positively predicted job stress (see Table 4). Perception of a caring ethical climate boosted the relationship between all the dark triad traits and stress. Among employees who scored less than average in perception of a caring ethical climate, the relationship between narcissism and psychopathy with stress was negative and became irrelevant in a group with higher-than-average results (see Table 5). Among workers who achieved less-than-average, average, and above-average outcomes regarding perceptions of a caring, ethical climate, a positive relationship was observed between Machiavellianism and stress, and it was stronger the more a caring ethical climate was perceived.

**Table 4:** Results of moderation analyses (*N* = 1,000).

Hypotheses	Moderating variable	Predictor	Coefficient	*t*	*p*	LLCI	ULCI
H1 (outcome: job stress)	Caring ethical climate	Narcissism	0.006	2.91	0.003	0.002	0.010
		Machiavellianism	0.009	3.75	0.000	0.004	0.013
		Psychopathy	0.010	3.93	0.000	0.005	0.016
H2 (outcome: exhaustion)		Narcissism	0.017	4.55	0.000	0.009	0.024
		Machiavellianism	0.021	4.88	0.000	0.012	0.029
		Psychopathy	0.023	4.82	0.000	0.014	0.033

LLCI = 95% confidence interval (low); ULCI = 95% confidence interval (high).

**Table 5:** Moderating effects of the dark-triad dimensions on job stress and exhaustion for different values of caring ethical climate as moderator (*N* = 1,000).

Caring ethical climate	Effect	*t*	*p*	LLCI	ULCI
−4.82 (−1 *SD*)	−0.058	−2.99	0.002	−0.096	−0.020
0.00 (*M*)	−0.029	−1.78	0.075	−0.060	0.003
4.82 (+1 *SD*)	0.001	0.01	0.991	−0.036	0.037
Narcissism as the independent variable and job stress as the outcome variable					
−4.82 (−1 *SD*)	0.055	2.19	0.028	0.005	0.105
0.00 (*M*)	0.099	4.46	0.000	0.055	0.143
4.82 (+1 *SD*)	0.143	5.75	0.000	0.094	0.191
Machiavellianism as the independent variable and job stress as the outcome variable					
−4.82 (−1 *SD*)	−0.097	−3.63	0.000	−0.150	−0.044
0.00 (*M*)	−0.046	−2.07	0.038	−0.089	−0.002
4.82 (+1 *SD*)	0.005	0.21	0.826	−0.043	0.053
Psychopathy as the independent variable and job stress as the outcome variable					
−4.82 (−1 *SD*)	−0.031	−0.90	0.364	−0.100	0.036
0.00 (*M*)	0.050	1.71	0.085	−0.007	0.107
4.82 (+1 *SD*)	0.132	3.93	0.000	0.066	0.198
Narcissism as the independent variable and exhaustion as the outcome variable					
−4.82 (−1 *SD*)	0.089	−1.96	0.051	−0.178	0.000
0.00 (*M*)	−0.013	0.33	0.278	−0.065	0.092
4.82 (+1 *SD*)	0.116	2.56	0.104	0.027	0.205
Machiavellianism as the independent variable and exhaustion as the outcome variable					
−4.82 (−1 *SD*)	−0.184	−3.82	0.000	−0.279	−0.089
0.00 (*M*)	−0.070	−1.77	0.066	−0.149	0.007
4.82 (+1 *SD*)	0.043	0.97	0.328	−0.043	0.130
Psychopathy as an independent variable and exhaustion as an outcome variable					

LLCI = 95% confidence interval (low); ULCI = 95% confidence interval (high).

Additionally, hypothesis 2, which posited that the perception of a caring ethical climate moderates the relationship between the dark triad personality traits and exhaustion, was confirmed. Narcissism, Machiavellianism, and psychopathy explained exhaustion in interaction with their perception of a caring ethical climate (see Table 4). In a group of workers who scored lower than average in perception of a caring ethical climate, psychopathy was negatively related to exhaustion, but narcissism and Machiavellianism were unrelated to exhaustion (see Table 5). In turn, among employees with a higher than average outcome regarding a perception of caring ethical climate, narcissism and Machiavellianism correlated positively with exhaustion, but psychopathy did not. For employees with average scores in their perception of a caring ethical climate, none of the three dark traits was significantly related to exhaustion.

Hypothesis 3, a positive relationship between job stress and exhaustion, was supported. Job stress positively predicted exhaustion (*b* = 71; *t* = 12.47, *p* = 0.000, *CI* [0.5987, 0.8222]). Among sociodemographic variables, only education level and sex were predictors of exhaustion, respectively (*b* = -21; *t* = -1.99, p = 0.046, CI [-0.4204, -0.0031]), and (*b* = 60; *t* = 3.57, *p* = 0.000, *CI* [0.2702, 0.9272]).

Moreover, hypothesis 4, which posited that job stress mediates the relationship between the dark triad personality traits and exhaustion and that this link is moderated by the perception of a caring ethical climate, amplifying this indirect positive relationship, was supported. The value of the moderated mediation index confirmed that narcissism is indirectly related to exhaustion and that this relationship is moderated by the perception of a caring ethical climate (0.0043, *SE* = 0.0002, 95% *CI* [0.0004, 0.0082]). The same situation was also observed regarding the two other manifestations of the dark triad as independent variables: Machiavellianism (0.0064, *SE* = 0.0021, 95% *CI* [0.0023, 0.0108]) and psychopathy (0.0075, *SE* = 0.0023, 95% *CI* [0.0030, 0.0123]).

Narcissism was negatively indirectly related to exhaustion through job stress, but only in a group of employees with lower-than-average results on perception of a caring ethical climate (indirect effect = −0.0413, 95% *CI* [−0.0767, −0.0077]). Among employees who scored average and higher than average in perception of a caring ethical climate, indirect effects of narcissism on exhaustion were irrelevant (indirect effect = −0206, 95% *CI* [−0.048, 0.0056] and indirect effect = 0.0001, 95% *CI* [−0.0316, 0.0310], respectively). Statistically significant positive indirect effects of Machiavellianism on exhaustion were observed in employees with average (indirect effect = 0.0697, 95% CI [0.0324, 0.1075]), and higher than average results in perception of a caring ethical climate (indirect effect = 0.1003 95% *CI* [0.0853, 0.1450]), but not in the group lower than average score (indirect effect = 0.0390, 95% *CI* [−0.0049, 0.0828]). In the group of employees who had lower-than-average scores in perception of a caring ethical climate, the negative indirect effect of psychopathy on exhaustion significant (indirect effect = −0.0683, 95% *CI* [−0.1153, −0.0256]) was found, in comparison to insignificant effect in the group with average scores (indirect effect = −0.0322, 95% *CI* [−0.0286, 0.0022]) and higher than average scores (indirect effect = 0.0038, 95% *CI* [−0.0353, 0.0425]).

## Discussion

This study aimed to investigate the relationship between the dark triad personality traits and burnout and to examine the role of job stress and perception of a caring ethical climate in this connection. Previous studies have presented an inconsistent picture, failing to account for the interactive effect of personality and workplace ethical environment in shaping the burnout phenomenon. This study's outcomes, designed to fill this gap, supported the presence of mechanisms underpinning the link between narcissism, Machiavellianism, and psychopathy with exhaustion. In line with assumptions, the harmful role of the perception of a caring ethical climate in relation to job stress and burnout among employees with dark triad personality traits was supported. According to the results, only one dark triad trait, Machiavellianism, predisposes an employee to experience stress, but none is related to burnout. On the other hand, a caring ethical climate was identified as a potential protection against stress and exhaustion; however, when interacting with dark triad traits, its harmful effects were noted.

These findings emphasize the negative consequences of employee–organization misfit. In this particular case, the perception of a caring ethical climate by employees who scored higher on dark triad personality traits evoked the need to adapt to ethical conditions that were inconsistent with their own ethical code of conduct, rules, and values. This requires putting in a great deal of effort to meet organizational ethical standards, leading to tension, dissatisfaction, inauthenticity, and frustration. This research showed that the consequences of being in this kind of situation may be job stress and exhaustion. When analyzing these phenomena, it is important to emphasize the potential differences observed regarding particular dark triad traits. Narcissism and psychopathy were negatively related to job stress at the low level of the perception of caring climate, and decreased to insignificance with the stronger perception of ethical climate as caring. Machiavellianism, on the other hand, was positively correlated with stress in lower-than-average results of caring climate, and this relationship was stronger the more the ethical climate was perceived as caring. The above tendency did not occur in relation to exhaustion. Machiavellianism was not related to exhaustion at the very low level of the moderator (caring climate), but positively related at the high level of the moderator. A similar tendency was observed in reference to narcissism. It suggests that Machiavellianism, among all dark triad traits, is the most vulnerable to stress and exhaustion under the perception of a caring ethical climate. On the other hand, it also shows psychopathy as the most insensitive dark triad trait for the potential negative effect of interaction with the perception of a caring ethical climate on stress and exhaustion. Among employees with a higher level of psychopathy, a lower-than-average level of perception climate as caring protects from stress and exhaustion, but in a group with higher-than-average results of caring climate, psychopathy was irrelevant to stress and exhaustion.

This study finding aligns with Li et al.'s (2022) research, which reported that workers with Machiavellian inclinations in a collectivistic work climate experience discomfort and a sense of inauthenticity, ultimately leading to motivation to leave the organization. However, some research has shown that the fit between employees with higher levels of dark triad traits and their perceptions of ethical workplace standards that align with their values and needs yields beneficial outcomes (O'Boyle et al., 2012; Webster and Smith, 2019). Apart from P-O fit theory (Kristof-Brown et al., 2005), the trait activation theory (Tett and Burnett, 2003) provides an appropriate way to explain the achieved results. In line with this approach, workers who score higher on narcissism, Machiavellianism, and psychopathy observe an ethical social environment, searching for cues that lead to expected behaviors and are the point of reference for ethical attitudes. A caring ethical climate offers a standard of ethical behavior that they do not find acceptable and is inconsistent with their inclinations. The stronger their perception of this kind of ethical standard, the more pressure they feel to adapt and the more frustration they experience, which can facilitate job stress and burnout. Finally, social environment suggestions and recommendations to engage in desirable behavior favor cooperation over being competitive and being altruistic over selfish, causing them to feel they have to act against themselves to meet the requirements of the social environment.

Apart from the mechanisms that rely on the direct effects of dark triad traits on burnout, moderated by a perception of a caring ethical climate, mechanisms of indirect effects of dark triad traits through job stress on exhaustion, moderated by a caring ethical climate, were observed. The more employees who have a higher level of dark triad traits perceived the ethical climate as caring, the more stressed they were and, in turn, the more exhausted. The direction of these changes is the same for each of the dark triad traits, but the negative consequences are the most powerful regarding Machiavellianism. Only in reference to this trait of character, average and higher-than-average results in perception of a caring ethical climate were positively related to exhaustion due to their higher level of job stress. In contrast, narcissism and psychopathy were not indirectly positively related to exhaustion via job stress, even in a group of workers with a higher-than-average perception of caring ethical climate. This shows that employees who score higher on Machiavellianism in comparison to employees who score higher on narcissism and psychopathic, bear the highest emotional costs of adapting to ethical standards that are contrary to their own. This corresponds with Spurk and Hirschi's (2018) study, where psychopathic and narcissistic inclined workers changed jobs according to their preferences for an organization with a competitive psychological climate, but workers with higher level of Machiavellianism, after switching jobs, did not perceive the organizational climate as competitive, which suggests that they believed in their adaptation abilities and influencing skills. Additionally, Bereczkei et al. (2010) confirmed that Machiavellianism at work is associated with aligning with social expectations and pretending to have altruistic attitudes, even if this requires the employee to behave in a manner inconsistent with their true preferences. On the other hand, as reported in this research, this effort to fit in can have negative emotional consequences for Machiavellian employees.

This study confirms the tendency of workers characterize by higher level of dark triad traits to fit their ethical behaviors to organizational requirements, even if doing so is contrary to their ethical values. Narcissists, due to their fragile and unstable self-confidence (Brookes, 2015; Geukes et al., 2017), are more prone to seeking social approval. Their primary motivation is the pursuit of being admired and viewed as unique, which serves to protect a grandiose self-view. Apprehension of social rejection (Besser and Priel, 2010) can motivate them to behave in a way that they do not prefer and that require significant effort.

These outcomes imply that employees with a psychopathic inclination are also sensitive to signals from the social environment regarding acting in an ethically suitable way as determined by the organization, despite their natural tendency to unethical behavior. In Wnuk's (2025) study, the fit between psychopathic characteristics and an instrumental ethical climate led to a more selfish approach at work as they felt they had permission to engage in moral behaviors that were in harmony with their nature because these behaviors were consistent with the ethical requirements of the organization. In the present study, they had to adapt to ethical standards that were contrary to their preferences, which evoked feelings of stress and exhaustion. Past studies have cast doubt on the susceptibility of individuals with psychopathic characteristics to change their attitudes under the influence of external sources caused by their limitations in impulsivity control and lack of sensitivity to punishment (Jones and Neria, 2019).

The main contribution of this study to the burnout research area and the personality and organizational psychology research areas is the role of perceptions of a caring climate as a facilitator of exhaustion among employees who score higher on dark triad traits. In addition to the moderating mechanism, moderated mediation was identified, showing that perceptions of a caring climate can amplify the indirect positive relationship between dark triad traits and exhaustion via job stress.

Practical implications of this study need to be addressed. First, human resources workers and representatives responsible for recruiting and hiring employees should ensure that candidates' personalities and their ethical norms and values align with the ethical standards implemented in the organization. To achieve this aim, companies should consider using psychometric tools to measure the dark triad traits of personality in the selection process, thereby effectively aligning personality predispositions and ethical values with the organization's ethical code of conduct. This mutual ethical fit will prevent individuals with dark triad traits from experiencing job stress and exhaustion. Earlier research has indicated beneficial outcomes resulting from such a matching between the dark triad traits and the work environment (O'Boyle et al., 2012; Webster and Smith, 2019). Additionally, workers characterized by dark triad traits in companies with a caring ethical climate should receive support in preventing and dealing with job stress and exhaustion in the form of workshops and therapeutic interventions.

### Limitations and future research

This study is limited by several factors. First, it was conducted among employees who were Polish citizens and were working under an employment contract in Poland. Future research should be conducted in another cultural context, above all one that is more diversified in terms of religious affiliations. Poland is a homogeneous society regarding religious denomination, where the vast majority of believers are members of the Roman Catholic Church. An ethical climate that is antagonistic to a caring ethical climate, such as an instrumental one, should be used because it matches workers with dark triad traits (Wnuk, 2025) and can protect them from stress and burnout. Within this ethical standard, they may feel comfortable and behave in an authentic and coherent way.

For measuring stress to reflect the multifaceted nature of this phenomenon, a more complex measure than a one-item measure is needed. This kind of tool has certain disadvantages: it cannot calculate reliability, often produces higher measurement error, and has low content validity, failing to capture the full breadth of a complex construct (McIver and Carmines, 1981). Also, using only negatively worded questions (items) regarding exhaustion would be associated with methodological flaws, including participants' difficulty with interpretation, increased risk of mistakes, and a lack of mirror-image responses (Zeng et al., 2024). It can lead to lower discriminant validity and reliability, which was not supported in this research, as the exhaustion measure demonstrated good reliability, as evidenced by Cronbach's alpha and composite reliability.

It is important to emphasize that methods used to examine common method bias have some weaknesses (Podsakoff et al., 2003). On the other hand, during the study preparation, the author took care to avoid this bias by mixing items and using control answers in the questionnaire. Additionally, the company specializes in this type of research and conducted this study, which implemented a mechanism that rejected participants' responses that were provided too quickly. Finally, the lack of a very strong correlation between research variables (more than 0.9) was an additional suggestion for the lack of common method bias problems.

Other research models with different configurations of variables were not tested, which means that it is not clear whether the dark triad personality traits are antecedents of stress and exhaustion or are in an inverse relationship. It is possible that stressful situations in the workplace and feeling exhaustion can favor the manifestation of dark triad personality traits. Only future longitudinal studies can confirm cause and effect because a cross-sectional design can only present the direction between study variables with some probability. Also, two types of narcissism should be taken into account, grandiose and vulnerable, as they are overlapping but different manifestations of this phenomenon (Miller et al., 2012). It cannot be ruled out that these two types of narcissism, interacting with the perception of a caring ethical climate, may explain job stress and exhaustion in different ways.

## Data Availability

The raw data supporting the conclusions of this article will be made available by the authors, without undue reservation.
